# Noble gas isotopes reveal degassing-derived eruptions at Deception Island (Antarctica): implications for the current high levels of volcanic activity

**DOI:** 10.1038/s41598-022-23991-3

**Published:** 2022-11-15

**Authors:** Antonio M. Álvarez-Valero, Hirochika Sumino, Antonio Caracausi, Antonio Polo Sánchez, Ray Burgess, Adelina Geyer, Javier Borrajo, José A. Lozano Rodríguez, Helena Albert, Meritxell Aulinas, Elena Núñez-Guerrero

**Affiliations:** 1 Departamento de Geología, Universidad de Salamanca, Salamanca, Spain; 2 Research Center for Advanced Science and Technology, University of Tokyo, Tokyo, Japan; 3 Sezione di Palermo, Istituto Nazionale di Geofisica e Vulcanologia, Palermo, Italy; 4 Department of Earth and Environmental Sciences, University of Manchester, Manchester, UK; 5 Geosciences Barcelona, CSIC, Barcelona, Spain; 6 Department of Physics, Engineering and Medical Radiology, University of Salamanca, Salamanca, Spain; 7 Instituto Español de Oceanografía, Centro Oceanográfico de Canarias, Santa Cruz de Tenerife, Spain; 8 Departamento de Mineralogía, Petrología y Geología Aplicada, Universidad de Barcelona, Barcelona, Spain

**Keywords:** Geochemistry, Volcanology

## Abstract

Deception Island is one of the most active volcanoes in Antarctica with more than twenty explosive eruptions in the past two centuries. Any future volcanic eruption(s) is a serious concern for scientists and tourists, will be detrimental to marine ecosystems and could have an impact to global oceanographic processes. Currently, it is not possible to carry-out low and high frequency volcanic gas monitoring at Deception Island because of the arduous climatic conditions and its remote location. Helium, neon and argon isotopes measured in olivine samples of the main eruptive events (pre-, syn- and post caldera) offer insights into the processes governing its volcanic history. Our results show that: (i) ascending primitive magmas outgassed volatiles with a MORB-like helium isotopic signature (^3^He/^4^He ratio); and (ii) variations in the He isotope ratio, as well as intensive degassing evidenced by fractionated ^4^He/^40^Ar^*^ values, occurred before the beginning of the main eruptive episodes. Our results show how the pre-eruptive noble gas signals of volcanic activity is an important step toward a better understanding of the magmatic dynamics and has the potential to improve eruption forecasting.

## Introduction

Understanding magmatic processes at depth is critical for the challenge of being able to confidently predict volcanic eruptions. A key component is the ability to monitor and interpret degassing processes within the magmatic plumbing system, as degassing often acts a prelude to major eruptions (e.g.^1,2^).

Noble gas isotopes represent versatile tools, potentially providing a means to decipher the origin and evolution of Earth’s materials due to their chemical properties (e.g., inert gases) and distinctive isotopic compositions for different geochemical reservoirs. In addition, they are particularly useful for tracing subvolcanic processes as their elemental ratios (e.g., ^4^He/^40^Ar) are often fractionated by the magmatic processes transporting them from depth to surface, such as melting, crystallization and degassing (e.g.^3–5^).

The geochemical information contained in subvolcanic volatiles is one of the cornerstones (together with seismicity and deformation) in monitoring active volcanoes and allows a modern understanding of the processes controlling the magmatic evolution at depth and related degassing mechanisms (e.g.,^2,6–9^). The information obtained from noble gas studies can be implemented into volcanic hazards assessment because magma dynamics in the volcanic plumbing systems and injection of fresh and undegassed magmas into subvolcanic reservoirs are key mechanisms triggering an eruptive event (e.g.,^10^). Geochemical studies have demonstrated that the arrival of the deep magma into shallower crustal levels can be recognized by increases in He isotope ratios (^3^He/^4^He) prior to the start of an eruptive event (e.g.,^6,7,11,12^). Recently, the monitoring of He isotopic ratios in active volcanic systems has provided quantitative information on the rate of magma input and volume change in the deep chamber preceding eruptions: on a timescale of months at Etna, Italy^2,3^; and over a longer period of ca. 10 years at Mt. Ontake, Japan^7^.

**Figure 1:**
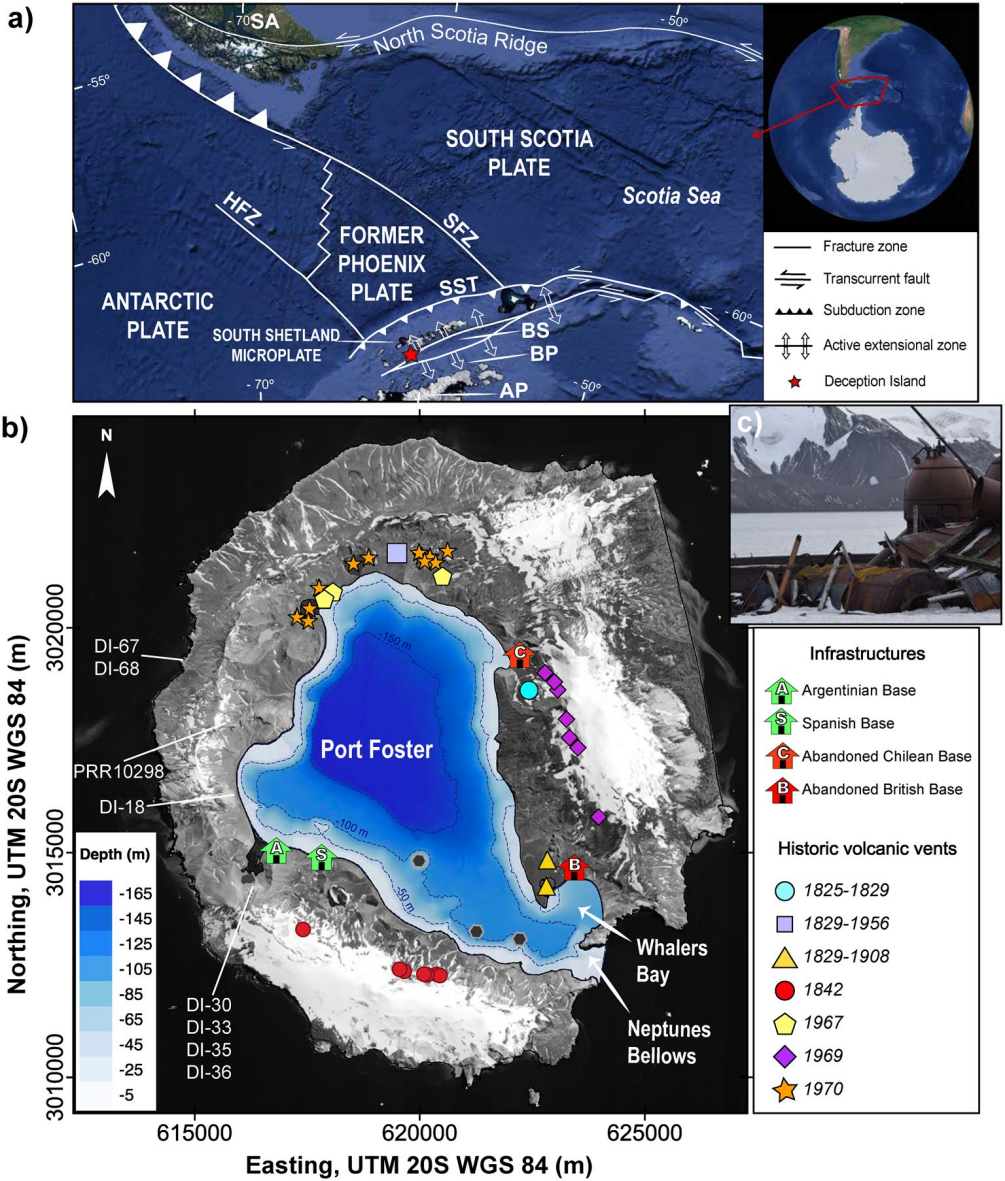
(**a**) Simplified regional tectonic map and location of the South Shetland Islands (modified from Martí et al.,^17^). AP: Antarctic Peninsula, BP: Bransfield Platform, BS: Bransfield Strait, HFZ (Hero Fracture Zone), SFZ (Shackleton Fracture Zone), SST: South Shetland Trench. (**b**) Deception Island orthophotomap (data obtained from Spatial Data Infrastructure for Deception Island SIMAC, Torrecillas et al. ^61^) and location of the studied samples. Existing and abandoned scientific stations are: *BAD* Base Antártica Decepción (Argentinean Scientific base), *BEGC* Base Española Gabriel de Castilla (Spanish scientific base), *BS* remains of the British scientific base, *CS* remains of the Chilean scientific base. (**c**) Example of a recent abandoned scientific base after a post-caldera eruption. This figure was generated with QGIS software version 2.18 Las Palmas (available at: https://www.qgis.org). Final layout was obtained with Adobe Illustrator CC 2015.3.1 (Copyright © 1987–2016 Adobe Systems Incorporated and its licensors).

One of the prime aims in the study of volcanic processes is to upcoming eruptions over short- and long timescales. To achieve this, it is critically important to understand the timing, duration and extent of magma recharge and its degassing in a volcanic plumbing system. Deception Island (South Shetland Islands, Antarctica) (Fig. 1) represents an excellent natural laboratory for studying the degassing processes occurring at depth because recent eruptive activity (e.g.,^13–15^) at this volcano can be assessed in relation to its record of past eruptions (e.g.,^16–18^).

We present the first He isotopic measurements for basaltic samples from the major volcanic events that led to the formation of Deception Island. These data are combined with the extensive published petrologic studies (e.g.,^16–19^), to explore and correlate the observed variations of the He isotopic ratios at depth with the main eruptive episodes during the volcanic evolution of the island; i.e. the relationship between the released gases at surface with the gases in inclusions trapped in the olivines at calculated depths. In contrast to the most abundant volatiles in volcanic systems (H_2_O, CO_2_, S, Cl, F), noble gases present at trace concentrations do not thermodynamically affect any physical processes that may occur as the magma ascends from the mantle to surface. The results of this study support the potential for understanding the relationships between the main volcanic eruptions and their related magma sources. The overall approach is potentially valuable as part of a future multidisciplinary study of volcanic monitoring at Deception Island aimed at forecasting future eruptions on the island and their style.

In addition to the largest eruption documented in Antarctica during the Holocene that formed the caldera^16,17,34^, Deception Island has produced numerous eruptions over the past two centuries (e.g.,^20,21^), as well as unrest episodes in 1992 (e.g.,^22^), 1999^23,24^, 2014–2015^14,15,24^, and the alert level (yellow) of eruption during the Spanish Antarctic campaign 2020–2021^25^. The most recent activity has been interpreted to indicate that an upcoming eruption is highly probable (e.g.,^16,21,26^). The logistics for high frequency monitoring of volcanic gases (e.g., noble gases) at Deception Island is not viable given its remote and inhospitable location off the coast of the Antarctic Peninsula. Here, we use noble gas isotopic data from melt/fluid inclusions in phenocryst minerals from the different volcanic deposits and combine these with data obtained from intermittent sampling campaigns. We show that the isotopic information obtained from the phenocrysts is an excellent complement to the usual activities that may be applied during high frequency monitoring of remote volcanoes.

## Deception Island: geological setting and volcanic history

Deception Island is an emerged composite volcano, forming a horseshoe-shaped island 15 km in diameter. It is located next to the intersection between the Hero Fracture Zone and the southwestern end of the Bransfield Strait. The latter is an extensional basin that separates the South Shetland continental microplate from the Bransfield Platform^27,28^ (Fig. 1). The complex geodynamics of subduction and back-arc spreading has directly influenced the timing and composition of magmatism in the region^29–31^. This geodynamical scenario has resulted in volcanic activity being mainly concentrated in Deception, Penguin and Bridgeman Islands during the Quaternary^30,32^.

The volcanic evolution of Deception Island is characterized by caldera collapse at ca. 8.300–3.980 years before the present (BP) based on palaeomagnetic measurements^33^ and caldera-related tephra across Antarctica^34^. The island’s history comprises three main volcanic episodes: pre-, syn- and post-caldera with the syn-caldera collapse representing the main volcano-stratigraphic marker^18^. The pre-caldera stage (< 750 kyr ^35^) corresponds to seamounts that coalesced to form a subaerial volcanic shield^16^. The caldera formation episode (i.e., syn-caldera stage) is characterized by 10’s m thick pyroclastic density current deposits (Outer Coast Tuff Formation; e.g.,^16,17^). The post-caldera phase consists of volcanic deposits erupted from > 70 scattered small-volume (< 0.1 km^3^), monogenetic eruptive centers, including several in recent years between 1829 and 1970 AD (e.g.,^16,17,36^). The post-caldera eruptions are documented as explosive, hydrovolcanic in some cases, and VEI 2–3^16,21,37^, forming fissure-sourced scoria, lavas, as well as tuff cone and tuff ring volcanoes^38^.

Deception Island is comprised of volcanic deposits showing tholeiitic affinity with a similar compositional range for pre-, syn-, and post-caldera products (bulk-rock and glass) ranging from basalts to trachydacites and rhyolites (e.g.,^16,18,39^). The island is associated with the Bransfield Rift and shows a similar subalkaline magma source with the additional minor influence of a subduction component. The latter indicates a distinctive source from other South Shetland Islands magmas^16,39^, and is likely related to the high degree of partial melting at Deception Island^18^.

The main volcanic hazards expected in the island henceforth are considered to be related to explosive hydrovolcanic eruptions due to a rapidly ascending magma interacting with meteoric and glacial surface water, the aquifer or seawater (e.g.,^13,19^). The last historic eruptions have ejected ash, lapilli and bombs, subordinate dilute pyroclastic density currents and destabilization driven mass wasting (e.g.^16,21,26,37^). Olivine phenocrysts are commonly subhedral- to euhedral for all three eruptive episodes. For a full petrological description of the Deception Island materials see Geyer et al. (2019) and a summary in the Supplementary Material.

## Results

New deep magma input in shallower layers of a volcanic plumbing system generally results in increasing He isotope ratios in the volatiles that are emitted at the surface (e.g.,^2,3,7^). Regular collection of volcanic gases is often part of a broader regime used to monitor volcanic systems, and He isotope ratios in the gas samples have proven to be an effective indicator of progressive changes in these systems (e.g.,^4,7,12^). In contrast, He isotope ratios in the volatiles trapped in inclusions hosted in olivine crystals of the primitive magma (Table 1) define the highest ^3^He/^4^He ratio achieved by the magma at depth, which in turn is the feeder of each subsequent eruptive event. Hence, we compared the He isotope values from well-constrained past eruptions in Deception Island, with the recent values from fumaroles (ca. 100ºC) and hot springs (ca. 60 ºC) in 2006 and 2009^13,40^, to provide a means to evaluate the present-day magmatic activity deeper in the plumbing system.

**Table 1:** Isotopic data of noble gases in the inclusions hosted in olivine phenocrysts (uncertainties are 1σ) (h-cr: hydraulic crushing; b.b: below blank).

Sample (olivine crushed)	IGSN	Extraction	Weight (g)	^3^He/^4^He (R/R_a_)	Error	R/R_ac_	^40^Ar/^36^Ar	Error	^4^He/^20^Ne	^4^He/^40^Ar*	[^4^He] (10^–9^ ccSTP/g)	Error	[^20^Ne] (10^–11^ ccSTP/g)	Error	[^40^Ar] (10^–8^ ccSTP/g)	Error	Fractionated air ^40^Ar/^36^Ar	Error
PRR10298 (pre-caldera)	PRR010298	h-cr 30MPaX3	0.0244	8.13	1.29	8.14	302.22	1.38	546.37	25.37	43.6	2.20	8.0	3.69	12.2	0.70	298.0	2.92
DI-18 (pre-caldera)	IED110018	h-cr 30MPaX3	0.0771	7.21	0.64	7.23	298.47	0.53	90.10	6.20	9.3	0.49	10.3	1.10	5.3	0.50	290.0	3.49
DI-67 (pre-caldera)	IED110067	h-cr 30MPaX3	0.0662	8.66	0.96	8.67	311.26	6.04	1140.00	39.09	11.4	0.58	b. b		0.8	0.13	300.4	18.35
DI-68 (pre-caldera)	IED110068	h-cr 30MPaX3	0.0838	6.58	0.61	7.10	288.22	0.91	3.75	1.09	12.4	0.64	331.0	33.22	97.6	4.88	284.9	2.03
DI-35 (syn-caldera)	IED110035	crush (50hits)	0.1219	5.11	0.59	6.02	300.02	1.03	1.75	0.15	2.2	0.11	125.7	20.06	12.8	0.90	333.8	5.01
DI-36 (syn-caldera)	IED110036	h-cr 30MPaX3	0.1117	6.98	0.59	7.12	296.95	0.44	13.79	21.00	6.7	0.34	48.5	4.90	4.2	0.29	295.5	6.73
DI-30 (syn-caldera)	IED110030	h-cr 30MPaX3	0.0645	6.68	0.55	6.69	322.09	0.86	277.66	3.70	26.1	1.30	9.4	0.96	9.3	0.60	297.3	4.00
DI-33 (post-caldera)	IED110033	crush (50hits)	0.0850	8.58	1.01	10.13	333.33	1.32	1.87	0.003	0.7	0.04	36.1	3.95	12.2	0.80	302.50	5.04

Fractionated air or initial ^40^Ar/^36^Ar = (^38^Ar/^36^Ar_sample_ − ^38^Ar/^36^Ar_air_)/(^38^Ar/^36^Ar_air_) × 2  ×  ^40^Ar/^36^Ar_air_ + ^40^Ar/^36^Ar_air_.

**Figure 2:**
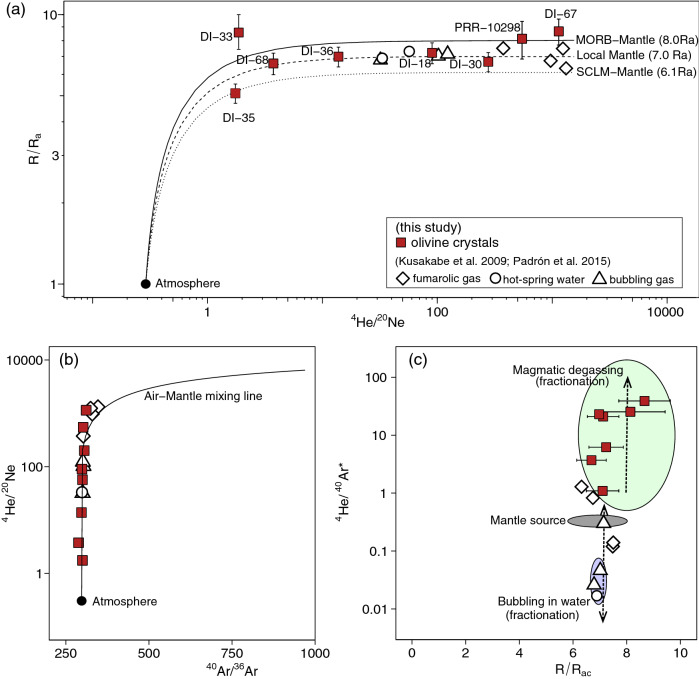
(**a**) ^3^He/^4^He versus ^4^He/^20^Ne diagram for Deception Island samples, showing mixing curves between low and high ^4^He/^20^Ne values of atmospheric, MORB and SCLM components (e.g.,^57^); (**b**) samples with the lowest ^4^He/^20^Ne values also have lowest ^40^Ar/^36^Ar values indicative of air contamination (see text and Table 1 for more details); (**c**) ^4^He/^40^Ar^*^ versus ^3^He/^4^He highlighting the mantle source area and the two main fractionation fields, i.e., magmatic degassing and bubbling in the waters (dashed arrows). Fumaroles and hot spring samples shift from the mantle source value (2–5) to (i) higher values due to magma degassing (as the olivines), and (ii) to lower values by fractionation during degassing from magma or dissolving in water followed by bubbling. Note that the noble gas isotopic ratios from the olivine crystals are plotted together with those from fumaroles and hot spring waters, which already represent fractionated noble gas elemental ratios.

Pre-caldera samples display ^3^He/^4^He ratios (R) in the range of 6.6–8.7 Ra (R_A_ = ^3^He/^4^He = 1.39 × 10^–6^) (n = 4), 5.1–7.0 R_A_ for the syn-caldera samples (n = 3), and 8.6 R_A_ for a post-caldera sample (n = 1) (Fig. 2a; Table 1). In comparison, the He isotope ratios of fumarole and hot spring samples are between 6.3 and 7.0 R_A_ (sampled in 2006;^13^), and 7.1–7.5 R_A_ (sampled in 2009^40^). Despite the slightly overlapping range of these data, they indicate that: (i) R/R_A_ values tended to increase between 2006 and 2009; (ii) pre- and post-caldera eruptions record the highest R/R_A_ values in the volcanic history of the island (Fig. 2a; Table 1); and (iii) the largest eruption (i.e. syn-caldera) was characterised by having lower He isotope ratios than pre-, post-caldera and in gases from fumaroles between 2006 and 2009.

^4^He/^20^Ne ratio is a sensitive tracer of atmospheric contamination and the results from the inclusions in the olivine phenocrysts show a wide range of values over several orders of magnitude from ca. 1.8 to 1100 (Fig. 2a; Table 1); all are significantly greater than atmospheric ratio (0.318). Hence, the correction for atmospheric He, based on ^4^He/^20^Ne, to the measured He isotope ratios (e.g.^41^) is minor (Table 1), and all sample have ^3^He/^4^He values within the range of mid-ocean-ridge-basalt (MORB) (Graham 2002, 8 ± 1 R_A_) or sub-continental-lithospheric-mantle (SCLM; 6.1 ± 0.9 R_A_; e.g.,^42^) (Fig. 2a). ^40^Ar/^36^Ar ratios (288–333) are within (or close to) the atmospheric ratio (298.5;^43^) displaying an air-mantle mixing trend when combined with the ^4^He/^20^Ne values (Fig. 2b). The ^40^Ar content of the samples can be corrected for an air contribution (^40^Ar^*^) as ^40^Ar^*^ = ^40^Ar – (^40^Ar/^36^Ar)_AIR_ × ^36^Ar, where ^36^Ar is assumed to be exclusively atmospheric-derived. The ^4^He/^40^Ar^*^ ratios in the olivine inclusions, range from c. 0.1 to 39 (Fig. 2c, Table 1), which extend to values higher than the mantle (about 1.2) and average crust production of about 5.

## Discussion

### Magma source beneath Deception Island

The magmas beneath Deception Island (e.g.,^16,18^) are the likely source of MORB-like ^3^He/^4^He signatures circulating within the volcanic plumbing system up to the surface (e.g., fluids in the magmatic chamber, fumaroles). In fact, the highest He isotopic ratios at Deception Island belong to pre-caldera samples and one post-caldera sample (> 8 R_A_) and are within the MORB range, whereas the syn-caldera samples are below this value, which may relate to the compositional variations of the different feeding magmas. The syn-caldera feeding magmas stalled at different depths within the crust during the longest period of the island’s history (from the pre-caldera eruptions at > 1 Ma). This allowed a higher crustal (radiogenic) contribution in the magmas lowering the usual MORB-like He isotopic ratio^18^. All the fumaroles’ samples have lower He isotope ratios than the highest pre-caldera value yet showing an increase with time from 6.3–7.0R_A_ (± 0.1) in 2006 to 7.1–7.5R_A_ (± 0.1–0.2) in 2009. This increase in the He isotopic values is coherent with the recent volcanic activity (ground deformation) retrieved on those dates^24^; and reflects changes in the magma dynamics such as the injection of deeper and less degassed pristine melts (e.g., for Deception Island^28^; for Mt. Etna^4^; and for Mt. Ontake^7^) that could feed an eruption.

Combining the ^4^He/^20^Ne and ^40^Ar/^36^Ar ratios we observed that volatiles in fluid inclusions, active fumaroles, hot-spring waters and bubbling gas at Deception Island show a mixing trend between a primitive MORB end-member and atmosphere (Fig. 2b). This is consistent with the petrologic model by ^18^ proposing a MORB mantle slightly modified by subduction as a source of magmas in Deception Island, although being aware that dissolved air in shallow-level groundwater could also have contaminated the magmatic gases during migration from the magma chamber to the surface.

The δD information in the phenocrysts, which is essential for understanding the origin of water from magma source at depth to its eruption in active volcanic systems, reveals that most of the Deception Island magmas^19^ match the range of the MORB mantle^44^ and other magmatic sources such as Koolau and Baffin Island considered to retain primitive D/H values^45^. In fact, the Deception Island magmas exhibit near invariant D/H values with variable extents of partial melting^19,46^. In summary, the He isotopic signal measured on the island’s surface reflect the pristine MORB origin of the magmas feeding the eruptions.

### Magma degassing at depth prior to pre-, syn- and post-caldera eruptions

Given that He is more soluble than Ar within silicate melt (e.g.,^49^), then magma degassing and water–gas interactions can modify the relative abundances of the noble gases in the phenocryst inclusions and in fumaroles and bubbling gases, respectively (Fig. 2c). Post magmatic solubility-controlled processes during aquifer interactions could also affect this ratio in the fumaroles (e.g.,^2,3,11^). Thus, noble gases in natural fluids emitted in active volcanic systems cannot be used as tracers of magmas ascent as they are modified during transport to surface. However, when the He ratios of the fluids are similar to the magmatic values they can indicate the main processes governing the chemistry of the magmatic fluids at depth.

Assuming the state of magma degassing is reflected by fumarole gases, the ^4^He/^40^Ar^*^ ratios for most of the waters and fumaroles in Deception Island (0.02–1.3^13^; 0.12–0.3^40^), are generally lower than the mantle and average crustal production ratios (i.e., 1–5; e.g.,^47,48^). If the ^4^He/^40^Ar^*^ variation resulted from fractionation during magma degassing, the residual volatiles trapped in olivine inclusions should have a higher ^4^He/^40^Ar^*^ ratio than the pristine volatiles in the magma (e.g.,^49^). However, olivine inclusions, mainly in the pre- and syn-caldera samples, show ^4^He/^40^Ar^*^ values up to 40 (yet with ^3^He/^4^He R_A_ of around 8, i.e., magmatic signal), which may reflect magma degassing episodes that led to the large eruptive events responsible for part of the island formation and the caldera’s collapse, respectively. Even if the higher ^4^He/^40^Ar^*^ values in the olivines are affected by the significant magmatic degassing, the hot-spring waters, fumarolic and bubbling gas samples could also have been affected by the degassed magma with a little fractionation occurring near the surface (lower ^4^He/^40^Ar^*^ values; i.e., < 1.3) (Fig. 2c).

Being aware that the relatively small dataset available may not provide a complete picture, the degassing suggested by the ^4^He/^40^Ar^*^ ratios and the MORB-derived He isotopic values (in both samples olivine phenocrysts at depth and hot-springs and fumaroles at surface), makes it possible to connect the three main degassing periods of Deception Island to their respective eruption episodes.

### Helium isotopic ratios as geochemical signal for the next eruption arrival

Eruptions at remote volcanoes can have widespread societal and environmental impacts as suggested by post-caldera tephra from Deception Island being present in distal marine sediments (> 500 km in distance, e.g.,^51,52^) and in ice cores (e.g.,^53,54^). Variations in ^3^He/^4^He values represent a key geochemical fingerprint for assessing the dynamics of generating batches of magma in a volcanic plumbing system and may enhance predictive capacity for an upcoming eruption (e.g.^2,3,11,12,50^). This geochemical signal complements the more commonly used geophysical signals (seismicity and ground deformation) to detect new magma inputs (and rates) that overpressure the chamber at depth. Since monitoring noble gases at high frequency is not routine at most active volcanoes, and not at all in remote locations, the ^3^He/^4^He variations measured within the olivine phenocrysts inclusions are critical for interpreting the past volcanic activity at Deception Island.

Kusakabe et al. (2009) reported δD and δ^18^O of the fumarolic fluids with values that ranged between seawater to local meteoric water (freshwater from crater lakes, ponds and glacier meltwater), which contrasts with the magmatic signatures of magmatic waters in the phenocryst inclusions^19^. However, ^3^He/^4^He values in the hot-spring and fumarolic fluids indicate MORB-like components. Therefore, in the fluids emitted on Deception Island^13^, even if the hydrogen and oxygen isotopic signal is very sensitive to the contamination of meteoric water, the He isotopes in the same emissions still maintain a magmatic signature, making them robust tracers of magmatic process at depth. Furthermore, He is very sensitive to mantle inputs that may have occurred in the absence of surface volcanic activity for years (e.g.,^4,7^). This implies that a possible new hydrovolcanic eruption at the island may follow a similar evolution to the last 1967–1970 eruptions ^18,21^, as well as fast magma ascent and quenching during eruption preserved in the inclusions, not only the magmatic He isotopic ratios but also the D/H magmatic isotopic composition (e.g.^18,19,45^).

**Figure 3:**
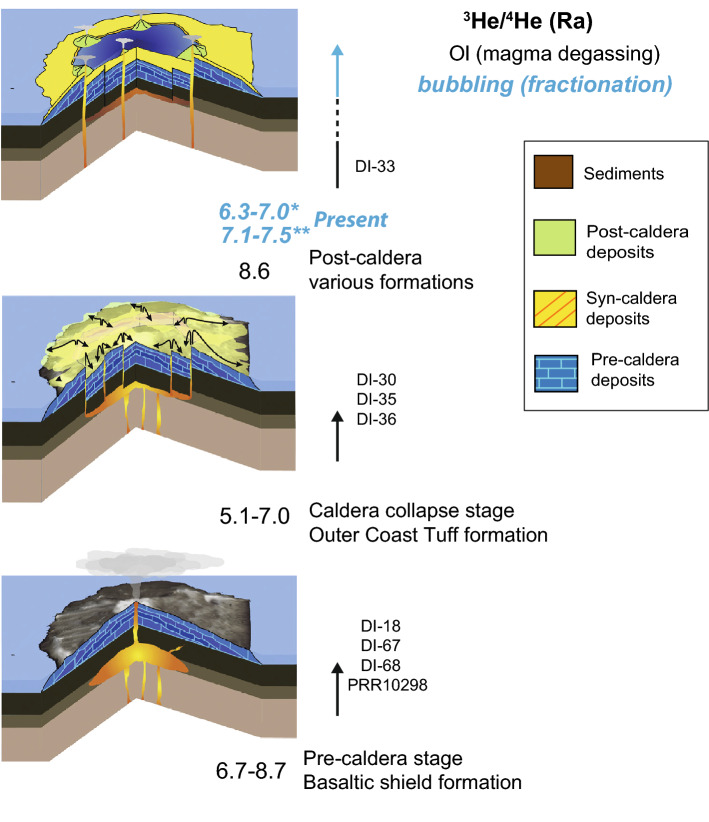
Summary-sketch evolution of the He isotopic ratios along the volcanic history of Deception Island (3D blocks are modified from ^17^) (*: Kusakabe et al.^13^; **: Padrón et al.^40^). This figure was generated with QGIS software version 2.18 Las Palmas (available at: https://www.qgis.org). Final layout was obtained with Adobe Illustrator CC 2015.3.1 (Copyright © 1987–2016 Adobe Systems Incorporated and its licensors).

The pre- and post-caldera eruptions were related to ^3^He/^4^He values of ca. 8 R_A_, whereas the syn-caldera event showed lower values of ca. 7 R_A_. Current He isotope ratios in the fumaroles and hot-spring waters measured over a period of three years show slight variations from 6.3–7 R_A_^13^ to 7.1–7.5 R_A_^40^, in line with complementary geophysical signals (e.g., ground deformation;^24^). Hence, by comparing noble gas signals in deep magmas and shallow waters (Fig. 3) our study could be considered as a complementary tool to reconstruct the evolution of the main volcanic phases during recent activity at Deception Island and to determine whether magmatic processes such as degassing are still active in the plumbing system. In addition, it may provide a useful means for future geochemical surveillance on the island, especially because of the lack of long-term data for noble gases, and the difficulty in retrieving new data at such a remote location. High frequency monitoring of the He isotopic ratios in the active hot spring sites on the island would be beneficial in detecting increases to 8–8.5 R_A_ values (as base line) and, therefore, provide a potential signal for an upcoming eruption.

## Conclusions

A key factor in planning for volcanic hazards is to understand the magma source at depth and the degassing processes in the plumbing system of the volcano. Our noble gas results indicate that the magmatic system beneath Deception Island released He with an isotopic composition within or near the MORB range values. Since recent He isotopic data at the fumaroles and hot spring waters also show a signal close to MORB ^13,40^, by linking data from the two sources provides insight into the magmatic dynamics in the Deception Island plumbing system (Fig. 3). For this purpose, the information given by noble gases trapped within inclusions in phenocryst is helpful for implementing the volcanic monitoring network as it offers information on the processes of magma degassing at depth.

The results of this study reveal that the surveillance of He isotope ratios provides critical data for helping to forecast future volcanic eruptions at Deception Island as has been found in other active volcanic areas elsewhere (e.g.,^7,11,12,55^). This study also suggests that the relationship between noble gas isotopes and eruptive episodes may provide an effective means of monitoring and predicting the behaviour of remote volcanoes that currently lack a high frequency monitoring network.

### Samples and analytical methods

Despite increased volcanic activity at Deception Island after caldera formation covered most of the pre- and syn-caldera deposits, we collected a wide suite of representative samples during several Spanish Antarctic Campaigns comprised between 2011 and 2018. We sampled different locations distributed over the entire island (Fig. 1), covering pre-, syn- and post-caldera materials (Table 1). We complemented our sampling with one additional rock sample from the Polar Rock Repository (PRR label in Table 1) (http://research.bpcrc.osu.edu/rr/). Aiming to make the results and sample archives of this work accessible to the broader community, all samples have been registered in the online System for Earth Sample Registration database (http://www.geosamples.org). Registered users can search the database to retrieve sample metadata and information about archived material.

Samples were crushed and sieved and hand-picked with tweezers to obtain mineral separates of crystals up to 5 mm size. The separated crystals were also inspected under a binocular microscope to ensure that they were free from any adhered matrix glass, and were ultrasonically cleaned using acetone, before loading into a crusher assembly used for noble gas extraction. Adsorbed atmospheric gases were pumped away during baking over night at 150 ºC before crushing extraction.

Noble gas analyses were carried-out using crushing extraction techniques in two ultra-high-vacuum mass spectrometers: (i) an MS-IV (modified VG-5400) in the Department of General Systems Studies, Graduate School of Arts and Sciences (University of Tokyo) for most of the samples (Table 1) by single-step hydraulic crushing which minimizes the release of matrix-sited components (e.g.,^56,57^). Details of the mass spectrometric technique and extraction/purification procedure at the University of Tokyo is described in ^58^; (ii) a Thermo-Helix-SFT in the Laboratorio de Isótopos Estables (University of Salamanca), with gas extraction by 50 strokes in an electromagnetic crusher.

Crushing analysis was undertaken to preferentially extract noble gases from inclusions (fluid and/or melt) in the olivine crystals. While it is possible that extreme crushing can release significant amounts of olivine matrix hosted gases (e.g.^59^), crushing experiments undertaken at both laboratories (same samples duplicated) gave similar results and are considered to be dominated by release from inclusions.

After each load of samples, we systematically ran crusher (empty) blanks before sample measurements and an additional calibration to ensure noble gas blank levels were low and the spectrometer’s sensitivity and tune settings were consistent. The HESJ (Helium standard of Japan^60^) and a calibration bottle containing air, were the standards used for He isotope analyses in Tokyo and Salamanca, respectively. Typical blank levels were generally below 1% of sample releases for He.

## Supplementary Information

Supplementary Information.

## Data Availability

All data analysed and generated during this study are included in this published article and its Supplementary Information file and archived at Zenodo (https://zenodo.org) a general-purpose open-access repository developed under the European OpenAIRE program.
