# Author Correction: Noble gas isotopes reveal degassing-derived eruptions at Deception Island (Antarctica): implications for the current high levels of volcanic activity

**DOI:** 10.1038/s41598-022-26369-7

**Published:** 2022-12-21

**Authors:** Antonio M. Álvarez-Valero, Hirochika Sumino, Antonio Caracausi, Antonio Polo Sánchez, Ray Burgess, Adelina Geyer, Javier Borrajo, José A. Lozano Rodríguez, Helena Albert, Meritxell Aulinas, Elena Núñez-Guerrero

**Affiliations:** 1 Departamento de Geología, Universidad de Salamanca, Salamanca, Spain; 2 Research Center for Advanced Science and Technology, University of Tokyo, Tokyo, Japan; 3 Sezione di Palermo, Istituto Nazionale di Geofisica e Vulcanologia, Palermo, Italy; 4 Department of Earth and Environmental Sciences, University of Manchester, Manchester, UK; 5 Geosciences Barcelona, CSIC, Barcelona, Spain; 6 Department of Physics, Engineering and Medical Radiology, University of Salamanca, Salamanca, Spain; 7 Instituto Español de Oceanografía, Centro Oceanográfico de Canarias, Santa Cruz de Tenerife, Spain; 8 Departamento de Mineralogía, Petrología y Geología Aplicada, Universidad de Barcelona, Barcelona, Spain

Correction to: *Scientific Reports* 10.1038/s41598-022-23991-3, published online 15 November 2022

The original version of this Article contained an error in the y-axis labels of Figure 2(c), where

“100000”

now reads:

“100”

“10000”

now reads:

“10”

“1000”

now reads:

“1”

The original Figure 2 and accompanying legend appear below.

**Figure 2:**
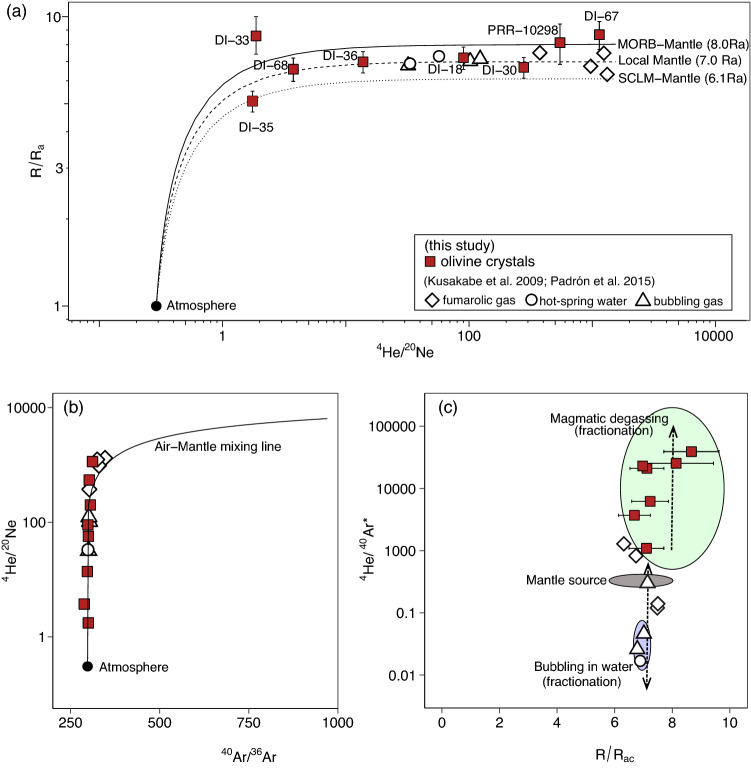
(**a**) ^3^He/^4^He versus ^4^He/^20^Ne diagram for Deception Island samples, showing mixing curves between low and high ^4^He/^20^Ne values of atmospheric, MORB and SCLM components (e.g.,^57^); (**b**) samples with the lowest ^4^He/^20^Ne values also have lowest ^40^Ar/^36^Ar values indicative of air contamination (see text and Table 1 for more details); (**c**) ^4^He/^40^Ar^*^ versus ^3^He/^4^He highlighting the mantle source area and the two main fractionation fields, i.e., magmatic degassing and bubbling in the waters (dashed arrows). Fumaroles and hot spring samples shift from the mantle source value (2–5) to (i) higher values due to magma degassing (as the olivines), and (ii) to lower values by fractionation during degassing from magma or dissolving in water followed by bubbling. Note that the noble gas isotopic ratios from the olivine crystals are plotted together with those from fumaroles and hot spring waters, which already represent fractionated noble gas elemental ratios.

The original Article has been corrected.

