# Supplementary material for: Noble gas isotopes reveal degassing-derived eruptions at Deception Island (Antarctica): implications for the current high levels of volcanic activity
Source: Sci Rep. 2022 Nov 15;12:19557. doi: 10.1038/s41598-022-23991-3 (PMC9666546; doi:10.1038/s41598-022-23991-3)
Supplement: Supplementary file 1 — Supplementary Information. [file 41598_2022_23991_MOESM1_ESM.pdf]

Noble gas isotopes reveal degassing-derived eruptions at Deception Island (Antarctica): implications for the current high levels of volcanic activity

Antonio M. Álvarez-Valero[1*], Hirochika Sumino[2], Antonio Caracausi[3], Antonio Polo Sánchez[1], Ray Burgess[4], Adelina Geyer[5], Javier Borrajo[6], José A. Lozano Rodríguez[7], Helena Albert[8], Meritxell Aulinas[8], Elena Núñez-Guerrero[1]

Summary of the petrologic features in the Deception Island samples

The **pre-caldera** samples mainly consist of lavas (mostly basaltic to basaltic-andesitic and basaltic-trachyandesitic) and hyaloclastite breccia clasts (see also [1]). These fragments show porphyritic texture of subhedral phenocrysts, mostly plagioclase and olivine hosting fluid/melt inclusions (Fig. S1; see also [2]) within a glassy groundmass dominated by plagioclase microlites and palagonitized scoria fragments. They also show thin, microlitic glassy margins with local palagonization, whereas the cores of larger fragments are widely unaltered. The selected pre-caldera samples are DI-18 and PRR-10298 (Table 1). Samples DI-67 and DI-68 are identified as crystal mush (plutonic) and syn-caldera, respectively[1,2], within syn-caldera deposits. Yet, since these syn-caldera deposits are next to pre-caldera materials in the west part of the island and their abundant olivine phenocrysts are restricted to juvenile domains with geochemical features similar to pre-caldera olivines [2], we are also considering these olivine samples (DI-67 and DI-68) as pre-caldera phenocrysts for this study.

The **syn-caldera** samples are a fine-grained welded ignimbrite consisting of juvenile scoria and lithics of mafic lava fragments, some of which are from the pre-caldera phase. These materials are embedded within a glassy, yellowish matrix with locally alteration to palagonite, similar to the pre-caldera samples, and phenocrysts of pyroxene, olivine and plagioclase. Syn-caldera samples used for his study are DI-35 and DI-36 (Table 1).

The **post-caldera** deposits are characterized by dark scoria fragments with minor lithic fragments. Juvenile pyroclasts show an aphanitic groundmass that contains crystals up to 10 vol% of the total rock volume. These are mainly microlites, needle-shaped laths of plagioclase and microcrystals (<< 1 mm) of euhedral clino- and orthopyroxene and rare olivine. Lava flow samples display a holocrystalline microtexture mostly consisting of plagioclase, olivine and opaque phenocrysts within a microcrystalline matrix. DI-33 is the studied the post-caldera sample (Table 1).

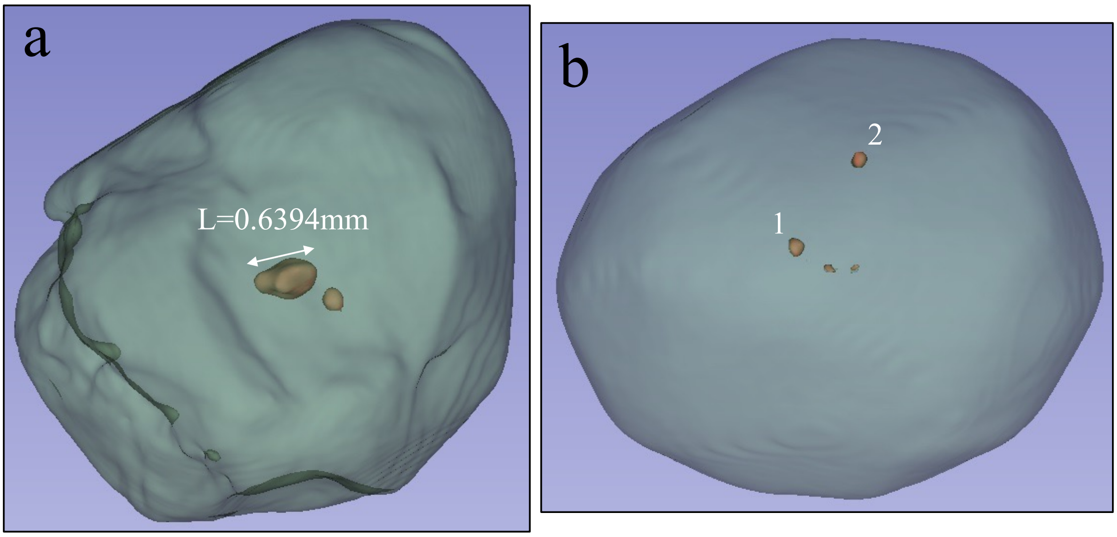

Fig. S1. Image of a micro-computed tomography (micro-CT) analysis to identify (non-destructive) the fluid/melt inclusions within olivine crystals: (a) sample DI-36, (b) (sample DI-35) before crushing them to extract their volatiles and determine the noble gas ratios. Note that the inclusions are not connected to any cracks in the mineral hosts.

Samples were imaged using a microtomograph Super Argus (SUINSA Medical Systems) at the University of Salamanca (NUCLEUS - USAL), to identify the presence of inclusions within the olivine crystals. The tomographic scans were performed at 50 kV and 900 µA (effective transverse and axial fields of view of 6.7 and 4.8 cm, respectively). Samples were set on a rotation stage, and transmission images were obtained for each 0.33° of rotation with a total of 1080 images (16 shots for each image). The isotropic pixel edge size (i.e. resolution) was 38 µm. Three-dimensional (3D) analyses were reconstructed from the transmission images by using "3D Slicer" software. L: diameter length. L=0.2474mm (small inclusion in olivine "a"); L=0.1337mm and L=0.1119mm (inclusions 1 and 2, respectively, in olivine "b").

References

1. Geyer, A. et al. Deciphering the evolution of Deception Island's magmatic system. *Sci. Rep.* **9**(1), 1-14 (2019).

2. Álvarez-Valero, A. M. et al. δD and $\delta^{18}O$ variations of the magmatic system beneath Deception Island volcano (Antarctica): implications for magma ascent and eruption forecasting. *Chem. Geol.* **542**, 119595 (2020).
